# Ferroptosis: The Initiation Process of Lipid Peroxidation in Muscle Food

**DOI:** 10.3390/antiox14101157

**Published:** 2025-09-24

**Authors:** Joseph Kanner, Adi Shpaizer, Oren Tirosh

**Affiliations:** Institute of Biochemistry, Food Science and Nutrition, Faculty of Agriculture Food and Environment, The Hebrew University of Jerusalem, Rehovot 76100, Israel; adi.shpaizer@mail.huji.ac.il (A.S.); oren.tirosh@mail.huji.ac.il (O.T.)

**Keywords:** ferroptosis, muscle cells, ROS, iron ions, lipoxygenases, hydroperoxides

## Abstract

Animal slaughtering causes the cessation of oxygen delivery and that of nutrients such as cystine, glucose and others to muscle cells. In muscle cells, the changes in oxygen level and pH cause mitochondria, the endoplasmic reticulum, xanthine oxidase and uncoupled NOS to increase the level of O_2_^•−^, affecting the generation of H_2_O_2_ and the release of iron ions from ferritin. The activation of enzymes that remove and dislocate fatty acids from the membrane affects the sensitivity of muscle cells to peroxidation and ferroptosis. Increasing PUFAs in membrane phospholipids, by feeding animals a diet high in w-3 fatty acids, is a driving factor that increases lipid peroxidation and possible muscle ferroptosis. The activation of lipoxygenases by ROS to Fe^3+^-lipoxygenase increases hydroperoxide levels in cells. The labile iron pool generated by a “redox cycle” catalyzes phospholipid hydroperoxides to generate lipid electrophiles, proximate executioners of ferroptosis. Ferroptosis in food muscle cells is protected by high concentrations of vitamin E and selenium. In fresh muscle cells, glutathione peroxidase (GSH-PX) and other endogenous antioxidant enzymes are active and prevent lipid peroxidation; however, muscle heating eliminates enzymatic activities, making cells prone to high non-enzymatic lipid peroxidation. In muscle cells, coupled myoglobin and vitamin E act as a hydroperoxidase, preventing the generation of lipid electrophiles. Free iron ion chelators or effectors such as deferoxamine, EDTA, or ceruloplasmin are strong inhibitors of muscle cell lipid peroxidation, proving that muscle ferroptosis is mostly dependent on and catalyzed by the labile iron redox cycle.

## 1. Introduction

Plants and animals after harvesting or slaughtering for food are organisms in the process of cell death. For years, a great mission for scientists was to explore the mechanism of cell death, since understanding it may open ways for extending life. Ferroptosis is a form of regulatory iron-dependent oxidative stress cell death, affecting membrane integrity by the generation of lipid hydroperoxides and cytotoxic advanced lipid end products (ALEs).

The initiation of lipid oxidation in biological systems at the chemical and biochemical level was divided many years ago into three steps: (1) the activation of oxygen to reactive oxygen species (ROS); (2) the oxidation of fatty acids by ROS and enzymes to hydroperoxides; and (3) the propagation of lipid hydroperoxides by auto-oxidation and iron-redox catalyzers to lipid free radicals, generating cytotoxic compounds [1]. All these steps were observed but not integrated into the unified cell molecular biology process known today, in part, as ferroptosis. Many factors connected to ferroptosis seem to affect lipid peroxidation in animal tissues after slaughtering; however, the most important are the cessation of oxygen delivery (ischemia/anoxia) and blood component transport to organs and cells. Ferroptosis is a process of regulatory cell death [2]. This unique cell death is derived by the obscure generation of lipid peroxidation products claimed to be the proximate executioners of ferroptosis. The specialized cell death program of ferroptosis is triggered at the end by insufficient activity of glutathione peroxidase 4 (GSH-PX4) [3].

## 2. Ferroptosis Inducers, in General and in Muscle Cells

The ferroptosis process, as known, involves an iron-dependent phospholipid peroxidation to cytotoxic compounds regulated by cellular pathways, including oxygen presence, redox changes, and activation of oxygen to ROS, iron handling, mitochondria activity/dis-activity, endoplasmic reticulum, and lysosomal effectors [4].

**The electron transport chain** (ETC), localized in the inner mitochondrial membrane, seems to act as the primordial generator of O_2_^•−^, H_2_O_2_, and HO^•^. In mitochondria, ETC-I and ETC-III are the main generators of superoxide [5]. Inhibition of ETC-I and ETC-III by rotenone and antimycin A suppresses cysteine deprivation-induced ferroptosis [4]. It seems that ETC-III dominantly plays a primary role in the execution of ferroptosis under conditions of nutrient starvation such as in muscle cells after animal slaughtering [6].

**Figure 1:**
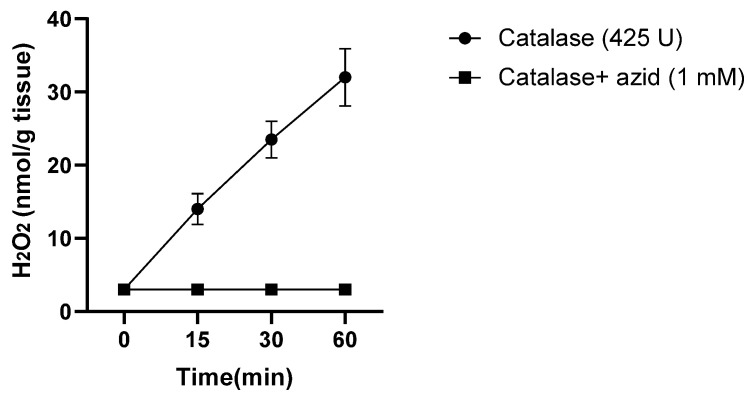
Generation of H_2_O_2_ by turkey dark muscle cells stored at 37 °C, determined by the peroxidase activity of catalase and oxidation of ^14^C-formate to ^14^CO_2,_ collected by KOH and detected by a scintillator. Adapted from Harel S. and Kanner J, 1985 [11].

**Ischemia and reperfusion** are two main phases responsible for muscle injury through an increased generation of ROS such as superoxide anion radicals (O_2_^•−^), hydrogen peroxide (H_2_O_2_) and hydroxyl radicals (HO^•^) that trigger oxidative stress-induced cell death. ROS in the muscles are mainly formed from primary sources such as NADPH oxidases (NOXs), redox changes affecting the mitochondria respiratory chain, and secondary sources of activation such as; xanthin dehydrogenase converting to xanthine oxidase and uncoupled nitric oxide synthases (NOSs) [7,8]. ROS could be generated also by oxygen reduction by labile ferrous ions to O_2_^•−^and H_2_O_2_ [1,9]. In muscle foods, the act of animal slaughtering causes cessation of oxygen to organs and decreases the amount of oxygen for tissues and cells. After death and rigor mortis, cutting the meat into small parts exposes its surface to oxygen and the generation of H_2_O_2_ and lipid peroxides [10,11,12] (Figure 1).

These effects resemble, in part, heart muscle ischemia/reperfusion injury effects [8]. Studies have suggested that heart muscle reperfusion-induced oxidative injury is dependent on critical events such as succinate accumulation in mitochondria during the ischemia stage and generation of superoxide O_2_^•−^; cessation of cells due to depletion of amino acids, especially cysteine/glutathione, and accumulation of glutamate. Induction of muscle oxidative injury is also dependent on dislocation of membrane phospholipids such as phosphoetanolamines (PE) and other fatty acids, mostly fatty acyls-arachidonoyl (AA) and adrenoyl (AdA), activation of lipoxygenase 15/12, and generation of lipid hydroperoxides. The hydroperoxides are broken down by the labile iron pool (LIP) to lipid-cytotoxic electrophilic compounds, affecting the integrity of the membranes [13]. Indeed, Ma et al. [14], by using multi-omics and chemogenomic approaches, demonstrated that ischemia triggers a specific redox reaction that induces the increased generation of arachidonic acid 15-lipoxygenase-1 (ALOX15). This enzyme acts during the reperfusion stage with the labile iron pool (LIP) to catalyze phospholipid peroxidation and the breakdown of hydroperoxides into ferroptosis signals.

**Figure 2:**
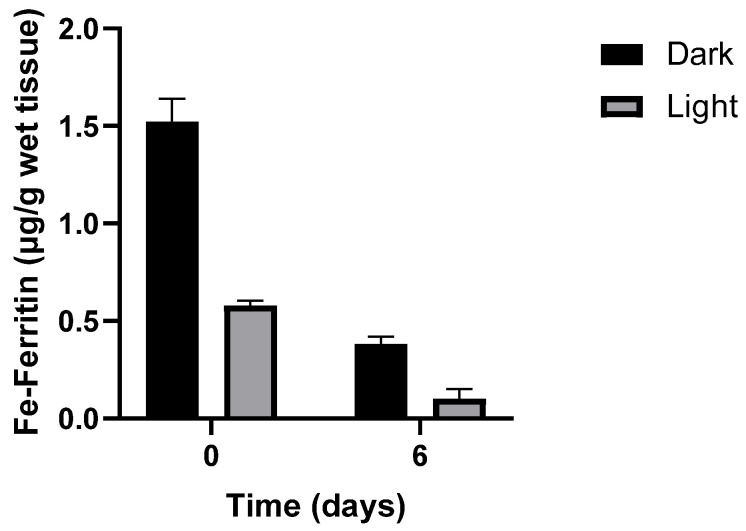
Loss of iron-ferritin isolated from dark and light turkey muscle after storage at 4 °C. Adapted from Kanner J. and Doll L. 1991) [19].

**Labile iron pool (LIP)**. Iron transferrin, after entering muscle cells through transferrin, is endocytosed. From endosome, iron is released and mediated by metal-reductase STEAP3 and divalent metal transporter (DMTI) to LIP [15]. Other major sources of LIP are ferritins from cytosol and mitochondria, stored as ferric ions as a complex of 24 subunits. Ferritin-bound iron is released into LIP by nuclear receptor coactivator 4 (NCOA4) dependent auto-phagosome degradation, activated during ischemia [16]. LIP could be released from ferritin through ferritin pores after reduction and solubilization of the ferric to ferrous iron within the ferritin core by reducing agents like ascorbic acid and superoxide anion radical [17] (Figure 2).

**Figure 3:**
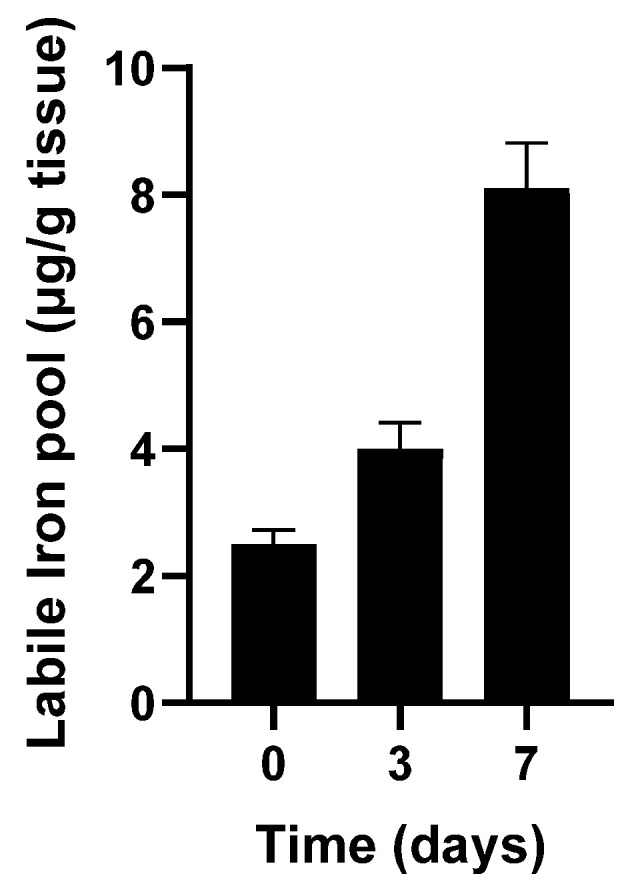
Catalytic free iron accumulated during storage of turkey dark muscle at 4 °C. Adapted from Kanner J et al., 1988 [20].

In the cytosol, labile iron is coordinated by a pool of small molecules (glutathione) and macro-molecules (iron-chaperones) that transfer the iron ions to different sites of the cell, and is affected by vitamin E [18]. A part of the LIP is an important precursor transferred to mitochondria for myoglobin biosynthesis. Previously, it was shown by us that after slaughtering, muscle cell ferritins are degraded, increasing the LIP in cells [19] Figure 3.

This LIP in muscle foods, in the presence of reducing compounds such as cysteine, glutathione, or ascorbic acid, acts as an “iron redox cycle”, initiating the Fenton reaction and lipid oxidation, generating hydroperoxides, and catalytically breaking down hydroperoxides into cytotoxic reactive aldehydes [9,20,21].

**Lipoxygenases** (LOXs) are non-heme iron-containing enzymes that play an important role in ferroptosis [22]. Electro-paramagnetic-resonance spectroscopic studies indicate that the native inactive form of the enzyme contains iron in its high-spin ferrous Fe^2+^state [23]. Lipoxygenase is activated to the ferric state by O_2_^•−^, H_2_O_2_, or previously formed hydroperoxides (LOOH) [1]. Lipid substrates for the enzymes are free fatty acids and phospholipids containing at least two non-conjugated, 1–4 carbon–carbon double bonds; because of that, oleic acid is not a substrate for lipoxygenases. The enzyme after activation could generate lipid allyl free radicals (L^•^) [14], which, in the presence of oxygen, generate a peroxyl radical LOO^•^; this radical is further reduced by the enzyme ferrous state to fatty acid hydroperoxides and back to the ferric active enzyme state. The hydroperoxides are not stable at 37 °C and break down into free radicals of LO^•^/HO^•^, inducing lipid auto-oxidation and the generation of many reactive carbonyls, electrophilic cytotoxic compounds, and cell death. Similar and most appropriate compounds are generated by the catalytic decomposition of lipid hydroperoxides by ferrous iron and heme proteins [1,14,24].

**Lipids and lipid metabolism** affect the sensitivity of ferroptosis. Free fatty acids can be taken into cells by diffusion and by protein transporters. Some lipids such as oleic acid, a mono-unsaturated fatty acid (MUFA), can be synthesized de novo in cells from palmitate by elongation and desaturation enzymes or from diet. The incorporation of MUFA into phospholipids is activated by acyl-CoA synthesized long-chain member 3 (ACSL3) and polyunsaturated fatty acids (PUFA) by ACSL4. Phospholipids can be remodeled by enzymes that remove/add fatty acids from/to membrane phospholipids by phospholipase A2 group and other acyltransferases, affecting the membrane sensitivity to peroxidation and ferroptosis. Increasing PUFA in membrane phospholipids is a driving factor for peroxidation and ferroptosis [22]; however, MUFA-enriched cells exerted no impact on these parameters [14]. A high fat-diet (HFD-PUFA) fed to rats, as an in vivo model of heart-muscle ischemia, had a significant impact on increasing lipid peroxidation parameters such as MDA and diminished GSH content, leading also to a significant increase in mortality [14]. In line with these results, our earlier report on feeding calves with PUFA found, after slaughtering, an increased susceptibility of the muscles to lipid peroxidation. Muscle and extracellular lipid peroxidation further increased with the addition of salt [25] or the inhibition of GSH-PX and other antioxidant enzymes [1,20,26,27].

## 3. Ferroptosis Prevention, in General and in Food Muscle Cells

### Antioxidant Enzymes

**Figure 4:**
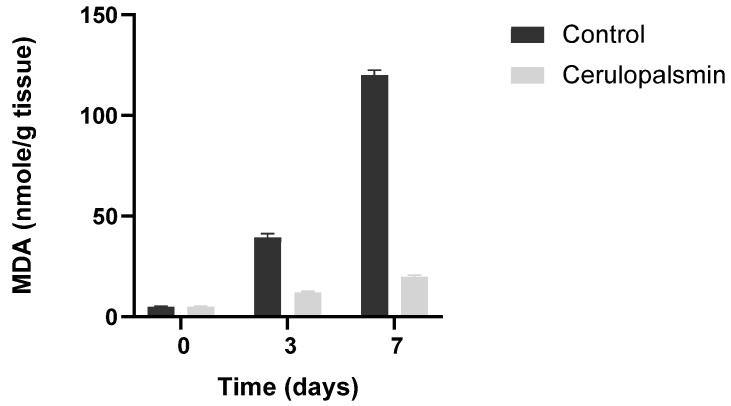
Ceruloplasmin (150 U/g tissue) inhibits in situ lipid peroxidation of minced turkey muscle tissues. Control, minced muscle 2- Minced muscle in the presence of ceruloplasmin. Adapted from Kanner J et al., 1988 [26].

Superoxide dismutase, catalase, glutathione peroxidase, ceruloplasmin, and peroxiredoxins are the main enzymes in biological systems, including muscle cells, to fight against ROS [1,28,29,30]. Anther factor in mitochondria that protects against ferroptosis is Coenzyme Q- (CoQ). CoQ can be reduced by dihydroorotate dehydrogenase to CoQH_2_, which, apart from transferring electrons to ETC III, can act as a local antioxidant, preventing mitochondrial and cell lipid peroxidation and limiting ferroptosis. CoQ is trafficked from mitochondria by a transfer protein (STARD7) to the plasma membrane, enhancing the cell's antioxidant defenses [31]. CoQ or CoQ•^.^ radical can be reduced back to CoQH2 also by the ferroptosis suppressor protein (FSP1) [32]. Ceruloplasmin, an antioxidant enzyme found in high concentrations in animal plasma, reduces ferrous ions to the ferric state, and by this, prevents the progression of lipid peroxidation in minced turkey muscle (Figure 4).

**Tetrahydrobiopterin (BH_4_)**, a natural nutrient, is biosynthesized and recycled by animal metabolism and also affects ferroptosis. BH_4_ acts as a cofactor for several enzymes such as nitric oxide synthase (eNOS) and, by generating ^•^NO, is involved in preventing ferroptosis. In BH_4_ deficiency, the uncoupled eNOS generates O_2_^•−^ interacts with ^•^NO to form peroxy-nitrite (HOONO), which decomposes into HO· and ^•^NO_2_, increasing ferroptosis [33].

**Antioxidants** are largely beneficial for human health [34]. Many natural and synthetic compounds with antioxidant action have been identified and are commercially available. **Selenium (Se)** is an effective modulator of the antioxidant systems in animals and humans. Selenium is involved in expression and synthesis of many seleno-proteins, including glutathione peroxidase (GSH-Px), thioredoxin (TrxR), and peroxiredoxins (PrxR). More than half of known seleno-proteins are directly or indirectly involved in antioxidant defenses and redox status maintenance. Selenium is the active center element in GPX4 enzyme, extremely important as an anti-ferroptosis enzyme. GSH-Px 1–4, including the membrane GSH-Px, are a family of antioxidant enzymes that decompose H_2_O_2_ and LOOH into non-radical compounds such as H_2_O and hydroxy-fatty acids, through the most effective anti-ferroptosis pathway [35]. GSH-Px acts as a very effective inhibitor of lipid peroxidation in fresh muscle foods [1,36]. However, processing of muscle foods by heating eliminates their activity as antioxidants, allowing the tissues to undergo high lipid peroxidation.

**Peroxiredoxin 6** (PRDX6), an important ferroptosis protector, is an enzyme acting as a phospholipid hydroperoxidase, but also as phospholipase A2 and lysophospholipid acyl transferase, an enzyme that can perform lipid detoxification and pathway repair by itself [37]. PRDX6 was found to have the ability to bind phospholipids and uses GSH as its physiological reductant.

**Myoglobin** (Mb) is known to catalyze the breakdown of lipid hydroperoxides into free radicals, a reaction that can enhance the propagation step and general lipid peroxidation [38,39]. During this reaction, a fraction of the free radicals are auto-reduced by metmyoglobin [40]. Our study demonstrated that metmyoglobin, at a rate concentration of ∼3:1 to hydroperoxides, acted anti-oxidatively and decomposed hydroperoxides, whose concentrations then remained at zero for a long time. This behavior has been recognized in the varying effectiveness of certain hemeproteins that are potent inhibitors at low peroxide levels, very weakly effective at high peroxide levels, and turn into oxidative catalyzers at very high peroxide levels [41]. Catechin, a known polyphenol, supports metmyoglobin antioxidation. The antioxidative activity of the complex metmyoglobin-catechin is stronger at pH 3.0 than at pH 7.0, indicating that this reaction is more dependent on metmyoglobin than on catechin. During this reaction, catechin or other polyphenols such as quercetin not only donate reducing equivalents to prevent lipid peroxidation but also prevent the destruction and polymerization of metmyoglobin. The results of this research highlighted the important and possible reactions of heme proteins and polyphenols as a redox couple, working as hydro-peroxidase or as pseudo-peroxidase. It was found also that d-α-tocopherol acts as a redox couple with metmyoglobin; at low pH, the couple is 150-fold more reactive than catechin [42]. Myoglobin is known to interact well with phospholipids and cell membranes. In tissues where myoglobin presents at high concentration, such as in the heart and muscles, during ferroptosis, it may act as a hydro-peroxidase using vitamin E or other reducing agents as redox cofactors.

Biochemical and tissue-level studies support the idea that Mb is also an important regulator of cellular nitric oxide (•NO) pools, in which Mb is a ^.^NO scavenger under normal cell oxygen levels, but also as a ^.^NO producer in anoxia [43]. Nitric oxide-myoglobin acts as an antioxidant in model systems of muscle lipid peroxidation [44,45], and in most probably cases, can act as an antioxidant in muscle cell ferroptosis.

**Vitamin E**. Vitamin E (*RRR*-α-tocopherol) is a potent anti-ferroptotic nutrient and the main chain-breaking lipid-soluble antioxidant in membrane cells, found in all animals but generated by plants. d-α-Tocopherol is a potent peroxyl radical scavenger that interrupts lipid peroxidation in membranes and lipoproteins by free radicals. The oxidized vitamin E is continuously restored by other hydrogen donors such as vitamin C and CoQH_2_, thereby oxidizing the latter and returning it to a vitamin E reduced state. If additional antioxidants are not present, the vitamin E phenoxy radical can reinitiate lipid peroxidation [46]. Tocopherol-quinone (α-TQ), generated by oxidation of α-tocopherol, can be reduced by reducing agents, NAD(P)H oxidoreductase enzyme, or ferroptosis suppression protein 1 (FSP1) to tocopherol-hydroquinone (α-THQ), a more efficient antioxidant than tocopherol. α-Tocopherol prevents RAS-selective lethal 3 (RSL3) induced ferroptosis cell death; however, the action of vitamin E hydroquinone is ~50-fold stronger. Vitamin E hydroquinone (α-THQ) is an endogenous regulator of ferroptosis, via redox control of 15-lipoxygenase, with very efficient protective activity, ~40-fold higher than that of d-α-tocopherol [23]. The catalytic activity of 15-lipoxygenase requires that the non-heme iron center be in the Fe^3+^ state; upon addition of α-THQ, a 50% decrease in the Fe^3+^ to non-active Fe^2+^ signal was measured. α-Tocotriene-hydroquinol (α-T3HQ), another natural antioxidant, reduced the ferric ion of lipoxygenase even more effectively. The potential of α-T3HQ as an anti-ferroptotic compound was found to be favorable for all ferroptosis measured tests and markers [23].

**Figure 5:**
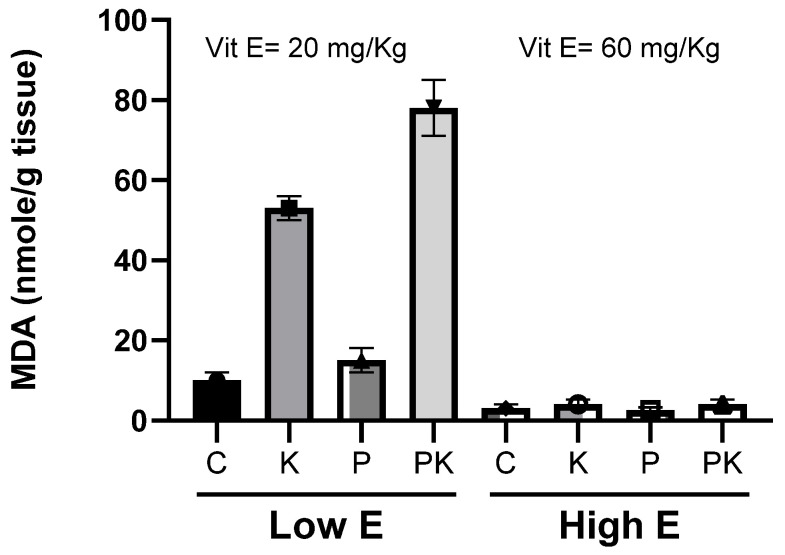
Lipid peroxidation and MDA accumulation during storage at 4 °C of longissimus dorsi muscle of calves fed diets supplemented with different amounts of vitamin E, with or without high PUFA content in feed:  control (C); vitamin E (E); PUFA in feed (P); kosher-salted (K); high PUFA and kosher-salted (PK). Adapted from Granit R et al. 2001 [25].

The effects of vitamin E supplementation [47,48], and high PUFA content in the feed, on calf muscle lipid peroxidation following NaCl addition after slaughtering and storage at 4 °C was studied previously by us [25]. The study found that even though the potential for peroxidation was enhanced by a diet rich in PUFAs or by the addition of NaCl, the high enrichment of muscle tissues with α-tocopherol almost completely inhibited muscle lipid peroxidation and oxymyoglobin oxidation [25], see Figure 5.

**Polyphenols** are a natural class of compounds found to protect against ferroptosis. Polyphenols were found to modulate iron metabolism and nuclear factor erythroid 2-related factor 2 (NRF2) signaling to inhibit ferroptosis [49]. Ferroptosis induced in piglet’s intestine by Diquat was inhibited by supplementing the animals with polyphenols extracted from *Ilex latifolia*, which contains high levels of phenolic acids and tannic acids [50]. Fisetin, another polyphenol, was found to ameliorate fibrotic kidney in mice via inhibiting ACSL4-mediated tubular ferroptosis [51]. Polyphenols were found by many authors to act as inhibitors of muscle food lipid peroxidation and were reviewed [32,52,53].

**Vitamins K** and **K**_2_ are well known for their canonical function as cofactors for γ-glutamyl carboxylase and generation of vitamin K-dependent protein factors for blood coagulation [54]. Vitamins K and K_2_ are redox-active quinones converted to corresponding hydroquinone (VKH_2_) by the vitamin K cycle. VKH_2_ was reported to be a potent radical trapping agent preventing lipid peroxidation [55]. FSPI, which reduces ubiquinone in the presence of NADPH, was found to also reduce vitamin K to VKH_2_, both acting as ferroptosis suppressor [56]. It is possible that supplementing animals and poultry with high vitamin K before slaughtering can improve protection against muscle lipid peroxidation, but such an experiment has never been performed.

**Nitric oxide** (^•^NO) is a free radical gaseous molecule with multiple biochemical and physiological functions [57]. The molecule is generated by enzymes in the nitric oxide (NOS) family, by converting arginine to citrulline using tetrahydrobiopterin (BH_4_) as a coupled factor liberating ^•^NO [58]. ^•^NO is also produced non-enzymatically by ferrous hemoglobin or myoglobin in the presence of nitrite [44,59,60],or from nitrite in stomach conditions (low pH and ascorbic acid) [61,62], NOS uncoupled with BH_4_ has a pro-oxidative activity because it generates superoxide radical and ^•^NO to produce peroxynitrous acid (ONOOH). Peroxynitrous acid is very unstable and decomposes into ^•^NO_2_ and HO^•^ radicals, both known as initiators of lipid peroxidation [63]. However, its pro-oxidative functions do not seem to play a role in ferroptosis [64].

**Figure 6:**
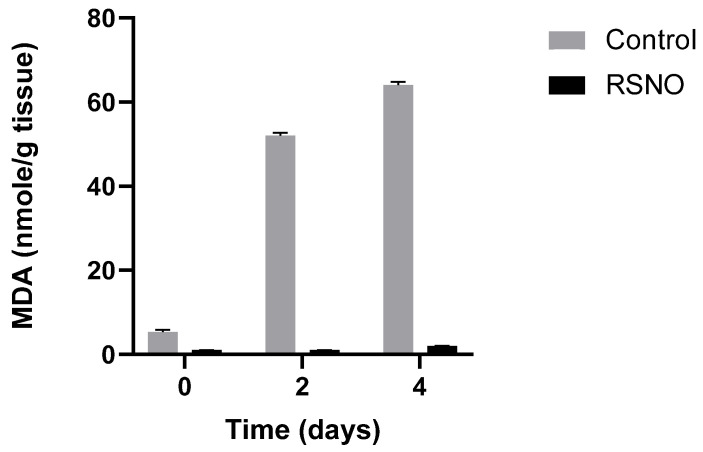
Lipid peroxidation and MDA accumulation in minced muscle, with and without the presence of NAC-SNO (RSNO), stored at 4 °C. Adapted from Kanner J et al., 2019 [61].

The role of ^•^NO as an antioxidant in food lipid peroxidation and in biological systems was first demonstrated by us [44,60,65]. The antioxidative role of NO/NOS in ferroptosis has been reported [66]. Our studies found that ^•^NO acts as an antioxidant, suppressing the Fenton reaction by scavenging HO^•^ radicals (in a reaction of “iron redox cycled” H_2_O_2_), but also (in a hydroperoxide-dependent lipid peroxidation) by scavenging LO^•^ and LOO^•^, to LONO and LOONO. ^•^NO is known also as an excellent ligand to ferrous-hemeproteins and iron complexes [67]. We found that both complexes act as very active antioxidants by rapid decomposition of H_2_O_2_ and LOOH into non-radical compounds such as HNO_2_ and LONO [59,60]. It was also demonstrated that ^•^NO, which acts as a very good ligand to iron, can inhibit enzymes such as lipoxygenase and cyclooxygenase, both containing iron ions at the active site of the enzyme [68,69]. N-acetylcysteine-NO (NAC-SNO) an ^•^NO donor, was found to replace nitrite in curing muscle foods as an anti-clostridial preservative, pigment developer, and antioxidant, almost without generating nitrosamines [61] (Figure 6).

**Figure 7:**
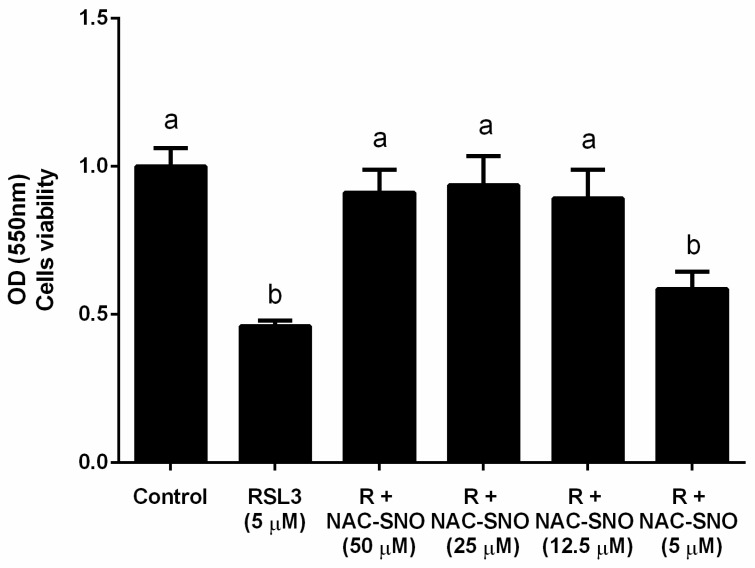
**The effect of NAC**^−^**SNO on cell viability in ferroptosis by RSL3**. Cell viability was measured by MTT assay of AML12 cells treated with 5 µM RSL3 and different concentrations of NAC-SNO for 6 h. a and b indicate significant statistical differences. Adapted from Shpaizer, A. PhD Thesis 2023 [70].

NAC-SNO at relatively low concentration (12 µM), was found to inhibit ferroptosis induced by RLS3 in a culture of hepatic cells. This effect was achieved most probably because of the biochemically broad antioxidant activity of ^•^NO [58,60,70], (see Figure 7).

**Synthetic antioxidants** that trap lipid radicals and suppress lipid peroxidation in ferroptosis are represented by ferrostatin-1 and liproxstatin-1 [71]. Ferrostatin-1 in the presence of ferrous ion acts similarly to GSH-PX4 by catalytic decomposition of lipid hydroperoxides with ~350-fold more activity than Trolox [72]. Butylated hydroxy toluene (BHT), a synthetic antioxidant known for preventing lipid peroxidation in foods, was found to act also as an inhibitor of ferroptosis in vitro and in vivo [73].

**Figure 8:**
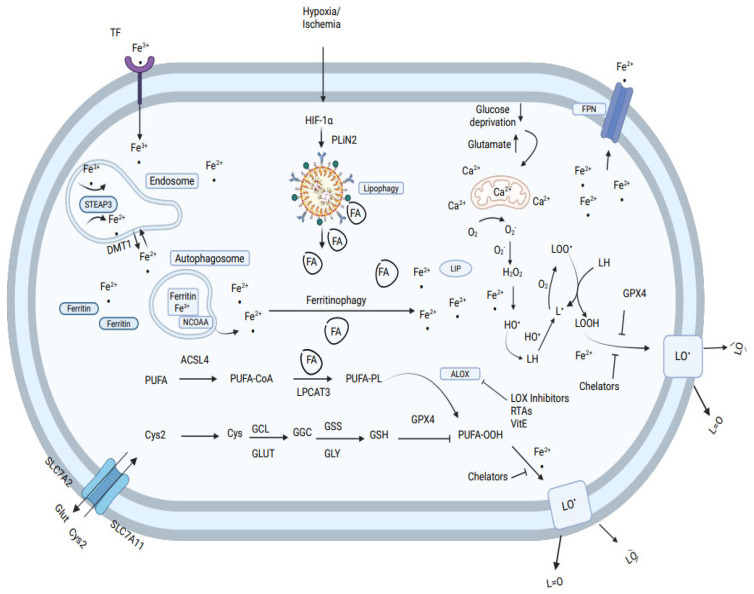
Molecular mechanism of ferroptosis in cells. Ferroptosis in muscle food is typically triggered by iron-dependent lipid peroxidation.

Animal slaughtering causes cessation of the flow of nutrients (cystine, glucose), oxygen depletion, and glutamate accumulation in cells. In muscle cells, the change in oxygen level, glutamate levels, and pH induce mitochondria to generate O_2_^•−^ and H_2_O_2,_ (see Figure 8). The release of iron from ferritin increases the labile iron pool (LIP) and is mediated by the endosomal metal-transferase STEAP3, divalent metal transporter (DMT1), and nuclear receptor coactivator 4 (NCOA4). Hypoxia inducible factor (HIF) system and perilipin 2 increase the amount of lipid droplets and lipolysis to fatty acid (FA) and metabolism of PUFA by long-chain-fatty-acid-CoA ligase 4, (ACSL4) and lysophospholipid acyltransferase 3, (LPCAT3) to membrane-rich phospholipids (PL), and PUFA, which is oxidized by arachidonic lipoxygenase (ALOX) to phospholipid hydroperoxides (PUFA-OOH). Activation of enzymes that remove or dislocate fatty acids from membrane affects the sensitivity of muscle cells to peroxidation and ferroptosis. Glutamate cysteine ligase (GCL) and glutathione synthetase (GSS) generate glutathione (GSH), which act as a redox couple with glutathione peroxidase (GSH-PX) for degradation of H_2_O_2_ and PUFA-OOH to H_2_O and the non-radical hydroxy fatty acids (LOH), preventing progression of lipid oxidation. Due to the cessation in the supply of cystine after slaughtering, GSH/GSH-PX couple activity decreases the antioxidative defense of muscle cells. The increase in PUFA in membrane phospholipids, by nutritional feeding, is a driving factor that increases lipid peroxidation and muscle ferroptosis. Food muscle cells are protected from ferroptosis by high concentrations of vitamin E, selenium, butylated hydroxy-toluene (BHT), and other radical- trapping antioxidants (RTA), and possibly by ferroptosis suppressor protein 1 (FSP1). Free iron ions chelators such as deferoxamine, EDTA, or ceruloplasmin (CP) act as strong inhibitors of muscle cell lipid peroxidation, proving also that muscle ferroptosis is mostly dependent on and catalyzed by the labile iron redox cycle.

## 4. Conclusions

Animal slaughtering causes cessation of the oxygen supply to the organs, decreases the level of oxygen in tissues and cells, and also causes cessation of the flow of nutrients such as glucose, cystine, BH_4_, and others to cells in muscle foods. After death and rigor mortis, cutting the meat into small parts exposes its surface to high oxygen level (ischemia/reperfusion)-induced generation of H_2_O_2_ and lipid peroxides. During muscle ischemia, the level of lactic acid increases, and the cell’s pH decreases, affecting the release of iron ions from ferritin. In muscle cells, the change in oxygen level and pH induces mitochondria, endoplasmic reticulum, xanthine oxidase and uncoupled NOS to increase the levels of O_2_^•−^ and H_2_O_2_ [74]. Both activate lipoxygenases, which act with the labile iron pool to catalyze phospholipid peroxidation. Ferroptosis in muscle food could be ameliorated by supplementing feed with increased levels of vitamin E and selenium, elevating GSH-PX4 and other protein selenium antioxidants, and also adding muscle food antioxidants such as: ^•^NO (NAC-SNO), polyphenols, and iron chelators (polyphosphates, EDTA, and ferrioxamine) [75]. We suggest, for the first time, that post-mortem metabolism, which is altered in the muscle environment after slaughtering, will dictate an imminent process of ferroptosis and that the lipid peroxidation rate within the muscle cells is the key factor for muscle food quality. Inhibition of this imminent ferroptosis rate by nutritional antemortem and post-mortem antioxidant treatments, within the muscle, is therefore essential for retaining food quality and human health. We hope that the review contributes to the possibility of fostering new scientific research connected to antemortem and post-mortem ferroptosis in muscle food.

## Abbreviations

ACSL4: long-chain-fatty-acid-CoA ligase 4, AIFM2: apoptosis inducing factor mitochondria associated 2, ALOX: polyunsaturated fatty acid lipoxygenase, ALEs: advanced lipid oxidation end products, AMPK: 5 ′-AMP-activated protein kinase, ACO1: aconitase1, ATF4: activating transcription factor 4, α-KG: alpha-ketoglutarate, CoQ: coenzyme Q, CoQH_2_: reduced CoQ, cPLA2: cytosolic phospholipase A2, CISD1: CDGSH iron-sulfur domain-containing protein 1, CP: ceruloplasmin, CA9: carbonic anhydrase 9, DMT1: divalent metal transporter 1, ETC: electron transport chain, FA-CoA: fatty acyl-CoA, FA-PL: 1-acyl phospholipid, FSP1: ferroptosis suppressor protein 1, Fe 2þ: reduced iron, Fe 3þ: oxidized iron, Fe–S cluster: iron sulfur cluster, FPN: ferroportin, FTH: ferritin heavy chain, FTMT: mitochondrial ferritin, FA-PL: 1-acyl phospholipid, yGCS: glutamate-cysteine ligase, GPX4: glutathione peroxidase 4, GRX: glutaredoxin, GSSG: glutathione disulfide/oxidized glutathione, GSH: glutathione, GS: glutathione synthetase, GLS: glutaminase, Gln: glutamine, Glu: glutamate, GLUD: glutamate dehydrogenase, GOT1: Glutamic-oxaloacetic transaminase 1 cytoplasmic, GPX4: glutathione peroxidase 4, ^•^OH: hydroxyl radical, PLOO ^•^ HO-1: heme oxygenase-1, HIF: hypoxia inducible factor, HILPDA: hypoxia-inducible lipid droplet-associated protein, HSPA5: heat shock protein family A member 5, Keap1: Kelch-like ECH- associated protein 1, LPCAT3: lysophospholipid acyltransferase 3, LIP: labile iron pool, Mfn2: mitofusin 2, miR: microRNA, MFRN: mitoferrin, MUFA: monounsaturated fatty acid, Nf2: merlin, NCOA4: nuclear receptor coactivator 4, Nrf2: nuclear factor erythroid 2-related factor 2, PRDX6: peroxiredoxin 6, PDSS: all trans-polyprenyl-diphosphate synthase, PUFA: polyunsaturated fatty acid, PLOH: hydroxy phospholipid, PLOO^•^: phospholipid hydroperoxyl radical, PLO^•^: phospholipid alkyl radical, PL^•^: phospholipid allyl radical, PLOOH: phospholipid hydroperoxide, PLIN2: perilipin 2, ROS: reactive oxygen species, RTA: radical trapping antioxidants, SLC7A11: cystine/glutamate transporter, SLC1A5: neutral amino acid transporter B, STEAP3: six transmembrane epithelial antigen of prostate metalloreductase, SCD1: stearoyl-CoA desaturase 1, TfR: transferrin receptor, TCA: tricarboxylic acid, Tf: transferrin, SLC7A11: cystine/glutamate transporter, YAP: Yes1 associated transcriptional regulator.
